# Monitoring of Nutrients, Metabolites, IgG Titer, and Cell Densities in 10 L Bioreactors Using Raman Spectroscopy and PLS Regression Models

**DOI:** 10.3390/pharmaceutics17040473

**Published:** 2025-04-04

**Authors:** Morandise Rubini, Julien Boyer, Jordane Poulain, Anaïs Berger, Thomas Saillard, Julien Louet, Martin Soucé, Sylvie Roussel, Sylvain Arnould, Murielle Vergès, Fabien Chauchard-Rios, Igor Chourpa

**Affiliations:** 1 Centre de Biophysique Moléculaire (UPR CNRS 4301), Département Nanomédicaments et Nanosondes, UFR de Pharmacie Philippe Maupas, Université de Tours, 31 avenue Monge, 37 000 Tours, France; 2 Ondalys, 4 Rue Georges Besse, 34 830 Clapiers, France; 3 Bioengineering, Bio-S, Technologie Servier, 905 Route de Saran, 45 520 Gidy, France; 4 Indatech—Chauvin Arnoux Group, 4 Rue George Besse, 34 830 Clapiers, France

**Keywords:** Raman spectroscopy, process analytical technology (PAT), CHO cell culture, bioprocess monitoring, chemometric analysis, IgG titer prediction, partial least squares (PLS) regression

## Abstract

**Background:** Chinese hamster ovary (CHO) cell metabolism is complex, influenced by nutrients like glucose and glutamine and metabolites such as lactate. Real-time monitoring is necessary for optimizing culture conditions and ensuring consistent product quality. Raman spectroscopy has emerged as a robust process analytical technology (PAT) tool due to its non-invasive, in situ capabilities. This study evaluates Raman spectroscopy for monitoring key metabolic parameters and IgG titer in CHO cell cultures. **Methods:** Raman spectroscopy was applied to five 10 L-scale CHO cell cultures. Partial least squares (PLS) regression models were developed from four batches, including one with induced cell death, to enhance robustness. The models were validated against blind test sets. **Results:** PLS models exhibited high predictive accuracy (R^2^ > 0.9). Glucose and IgG titer predictions were reliable (RMSEP = 0.51 g/L and 0.12 g/L, respectively), while glutamine and lactate had higher RMSEP due to lower concentrations. Specific Raman bands contributed to the specificity of glucose, lactate, and IgG models. Predictions for viable (VCD) and total cell density (TCD) were less accurate due to the absence of direct Raman signals. **Conclusions:** This study confirms Raman spectroscopy’s potential for real-time, in situ bioprocess monitoring without manual sampling. Chemometric analysis enhances model robustness, supporting automated control systems. Raman data could enable continuous feedback regulation of critical nutrients like glucose, ensuring consistent critical quality attributes (CQAs) in biopharmaceutical production.

## 1. Introduction

The metabolism of Chinese hamster ovary (CHO) cells during biopharmaceutical production is a complex and tightly regulated process involving numerous nutrients and metabolites [1]. Glucose, lactate, glutamine, and glutamate are central, interconnected components of this metabolic network. Indeed, glucose serves as the primary carbon source for energy production through glycolysis. However, excess glucose is often converted to lactate, which can inhibit cell growth if levels become too high. Glutamine plays a fundamental role in providing nitrogen for protein synthesis and replenishing the tricarboxylic acid (TCA) cycle. Glutamate, derived from glutamine, is another key player in the TCA cycle and amino acid synthesis. Real-time monitoring and understanding of the dynamics of these nutrients and metabolites are imperative for optimizing cell culture conditions, leading to increased efficiency and production yield of biotherapeutic proteins.

Raman spectroscopy has demonstrated significant potential for in situ, real-time quantification of critical nutrients, metabolites, titer concentrations, cell densities, and cell viability within mammalian cell culture [2]. Unlike near- and mid-infrared spectroscopy, Raman spectroscopy generates analyte-specific signals that do not overlap with water signals [3,4]. This makes it particularly advantageous for applications in cell cultures, where the matrix is predominantly aqueous [5]. Additionally, its compatibility with immersion probes makes Raman spectroscopy particularly well-suited for in-line bioprocess monitoring, as it is easy to implement, requires minimal maintenance, and enables high-throughput spectral acquisition without the need for extensive sample preparation [6].

Consequently, Raman spectroscopy presents significant advantages within the process analytical technology (PAT) framework by facilitating the real-time monitoring of bioprocesses and control of its critical process parameters (CPPs), such as nutrient levels, to ensure consistent product quality, defined as critical quality attributes (CQAs). By interpreting the correlation between cell culture environment (CPPs) and product quality (CQAs), PAT helps to identify and manage sources of variability, supporting more robust manufacturing processes and minimizing batch-to-batch variations. This is why the Food and Drug Administration (FDA) has been recommending the use of those methodologies in the pharmaceutical industry since 2004 [7].

Real-time analysis of Raman spectra relies on chemometric techniques to extract both quantitative and qualitative information about the process. In the context of chemometrics, Wold et al. [8] have clearly defined PAT into five distinct levels: determining quantitative or qualitative properties from spectra (Level 1), raw material quality checks (Level 2), batch process monitoring (Level 3), predicting product quality (Level 4), and implementing feedback control to ensure consistent quality (Level 5). To the best of our knowledge, most studies in the literature are focused on PAT Levels 3 and 4, with limited attention given to the dynamic feedback control of bioreactors during cell culture aimed at adjusting parameters to achieve the desired CQAs values.

Accordingly, works published over the last few decades have demonstrated the successful application of in-line Raman spectroscopy for real-time monitoring of substrates and metabolites within bioreactors. In 2011, Abu-absi et al. [9] were the first to report the feasibility of in-line Raman spectroscopy for the non-invasive, real-time monitoring of multiple components in mammalian cell culture bioreactors. Using partial least squares (PLS) modeling, the authors correlated Raman spectral data with concentrations of glucose, glutamine, glutamate, lactate, and ammonium, as well as VCD and TCD in 500 L bioreactors (300 L working volume). Subsequent studies further demonstrated the scalability and versatility of Raman-based monitoring. For instance, Whelan et al. [10], Mehdizadeh et al. [11], and André et al. [12] used Raman spectroscopy to quantify similar parameters at laboratory scales (<50 L), while Berry et al. [13] explored its application across larger scales, noting the challenge of cross-scale predictions due to scale-dependent variations. André et al. [14] developed global PLS models that predicted glucose and lactate concentrations across various cell lines, including mammalian and insect cells, demonstrating cross-cell-type applicability. Kozma et al. [15] advanced the technology by comparing Raman and NIR models, with Raman showing superior performance, allowing for rapid calibration and scaling from shake flasks to larger bioreactors. Further advancements include the use of Raman spectroscopy to control CQAs such as glycosylation and glycation, as highlighted by Li et al. [16] and Gibbons et al. [17]. Additionally, numerous studies, such as those by Rafferty et al. [18], have explored alternative chemometric modeling techniques like support vector machines (SVMs) and random forests (RFs), comparing their performance with traditional PLS models across multiple bioreactor scales. Machine learning approaches, such as those described by Tulsyan et al. [19,20], have integrated just-in-time learning models for real-time process monitoring, further enhancing model robustness and applicability across various scales. Recent work by Khodabandehlou et al. [21] combined deep convolutional neural networks (CNNs) with Raman spectroscopy to improve the prediction of glucose, lactate, and VCD concentrations by addressing challenges in baseline correction of Raman spectra. Recent advancements highlight the growing role of Raman spectroscopy in bioprocess control and optimization. Berry et al. [22] utilized Raman spectroscopy for feedback control of glucose, minimizing glycation through automated glucose regulation. Other studies have expanded this approach, utilizing Raman for controlling feeding strategies and metabolic regulation, including glucose, lactic acid, and pyruvate, to optimize product quality and process efficiency in both fed-batch and continuous biomanufacturing processes [23,24,25,26].

Despite the wealth of information available in the literature, issues remain. A key challenge is the development of analyte-specific calibration models, hindered by cross-correlation between analytes [27]. Additionally, the complexity of the Raman signal background evolves during cell culture, affected by fluorescence and light scattering. These factors distort the signal and can lead to baseline shifts and offsets [5], as well as loss of Raman bands. To address this issue, the use of chemometric techniques has shown potential in mitigating these effects and enhancing the reliability of the models.

The aim of this paper was to make a step forward toward industrial transposition of Raman spectroscopy for bioprocess monitoring, particularly using this innovative sensor to ensure real-time, non-invasive data collection. The data generation strategy implemented here enables the decoupling of spectral interferences and analyte concentrations, providing robust models for fed-batch CHO cell cultures. In the present study, the interest was to improve each model by a specific combination of wavenumber (variable) selection, data preprocessing, and model validation. The individual model optimization for each CPP is a strength of the study, which followed a pragmatic aim of industrially relevant implementation of Raman-based PAT, which is still very limited, while apparently feasible. In addition to the performance of each model, the present study was focused also on challenging its specificity, by acting on both the selection of specific variables and by using one batch with kinetics perturbed by cell death induced by nutrient lack. That could correspond to a real-world situation, where there is a need to recognize the deviation from other batches, all of which started under similar conditions. In addition, such a method is intended to mitigate model cross-correlation. Taken together, the described issues constitute the originality of this study.

We believe that valuable and specific prediction of the CPPs (using models optimized via a combination of all the available options) is a rational way to enhance the industrial attractivity of Raman-based PAT.

## 2. Materials and Methods

In the present paper, five cell cultures were carried out at the 10 L scale.

**Figure 1:**
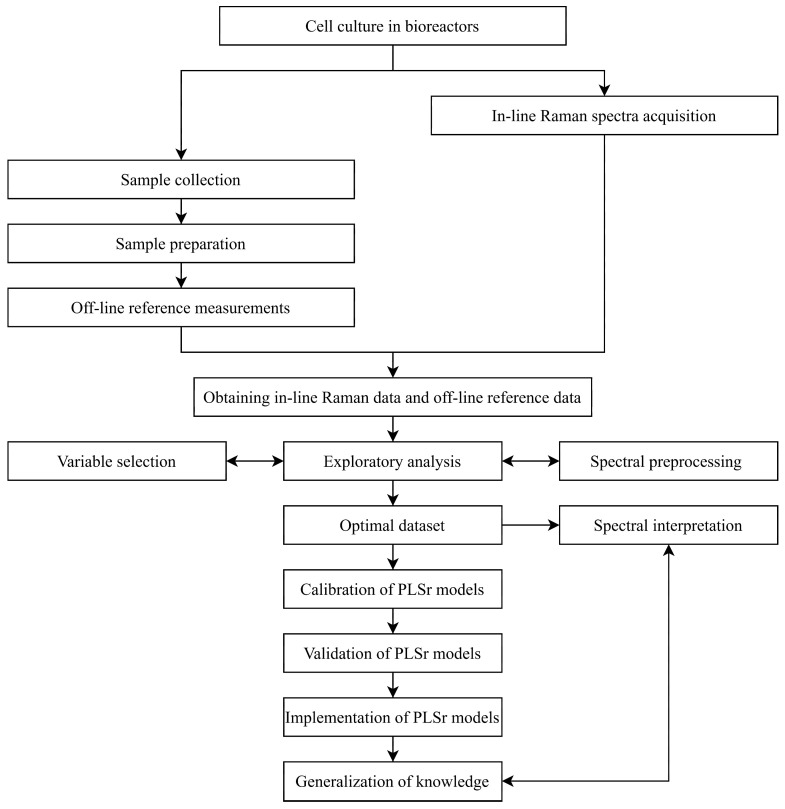
Flowchart of in-line Raman spectroscopic development for CHO cell culture monitoring.

Figure 1 presents a flowchart defining the general process of developing in-line Raman spectroscopic tools. The chart provides a chronological summary of all key steps. In brief, cell culture is initiated while the Raman spectrometer runs simultaneously for continuous in-line data acquisition. At regular intervals during cell culture, samples are manually collected from the bioreactor for off-line reference measurements. Once both in-line and off-line data are gathered, the chemometric analysis process begins. This involves cleaning outliers, optimizing chemometric processes (such as variable selection and preprocessing), and then calibrating and validating partial least squares (PLS) regression models.

### 2.1. Cell Culture: From Pre-Cultures to Bioreactors

A CHO-derived cell line producing an IgG1 monoclonal antibody (mAb) (Technologie Servier, Gidy, France) was thawed and expanded in Corning^®^ cell culture flasks in suspension, seeded at an initial density of 5 × 10^5^ viable cells/mL for 2 days of pre-culture. Pre-culture was carried out under controlled conditions, namely 37 °C, 85% RH, and 5% CO_2_ in a CHO-adapted serum-free basal medium supplemented with L-glutamine.

Following pre-culture, 10 L Eppendorf BioBLU^®^ 10c single-use bioreactors (Eppendorf France SAS, Montesson, France) were inoculated at a density of 6 × 10^5^ viable cells/mL at 70% of the final volume medium (7 to 10 L working volume), using the same medium as the pre-culture. The bioreactors were controlled via a Sartorius Biostat^®^ B-DCU II system, equipped with pH (Mettler Toledo, Viroflay, France), pO_2_ (Mettler Toledo, Viroflay, France) and PT100 temperature sensors, along with a pitched blade impeller set at 190 rpm. The cultures were maintained at 37 °C, with pH controlled by sparging CO_2_ or adding 1 M NaOH, and pO_2_ was adjusted through an O_2_ sparger. The feeding process was optimized through a mixture of Feed A and Feed B (10% *v*/*v* of Feed A) combined with 450 g/L glucose solution added once a day from day 2 to maintain a glucose concentration close to 5 g/L (±1 g/L) until the end of bioproduction. An exception was made for one cell culture, where feeding was stopped on day 10 to induce cell death. The objective of this specific experiment was to dissociate the cross-correlation of nutrients and metabolites.

The protocol of cell death induced by nutrient exhaustion (feeding stop) has been previously well-established in similar cultures of CHO. The main advantage of this method was to be close to a possible real-world situation, relevant to the industrial context. Other advantages were that this does not involve other reagents/factors and leads to a rather predictable effect on the cells and the produced antibody. The latter reasons were particularly important to not introduce new parameters in the models. Based on previous experience, the moment of feeding stop was chosen when the cell density has reached a plateau, (day 10) but not too late, to have enough time to observe the stages of the cell evolution upon cell death.

Foam was managed as necessary using appropriate antifoam agents. Bioreactor cell culture lasted 14–16 days and was harvested according to viability criteria. Sampling for off-line analysis began on day 0, with samples collected twice a day from day 2, immediately before and after feeding. All operations were meticulously performed outside the spectral acquisition periods.

### 2.2. Off-Line Reference Measurements

For the off-line reference measurements, a Beckman Coulter ViCell (Beckman Coulter, Roissy, France) was employed to assess cell density characteristics such as viable cell density (VCD) and total cell density (TCD). Concurrently, a Roche Cedex Bio HT (I&L Biosystems France SAS, Champagne-au-Mont-d’Or, France) was used to measure a range of biochemical parameters including glucose (Glc), glutamine (Gln), glutamate (Glu), lactate (Lac), and IgG titer. In short, according to the manufacturer, Glc, Lac, Gln, and Glu are measured using enzymatic assays. Glucose is phosphorylated by hexokinase (in the presence of glucose-6-phosphate dehydrogenase (G-6-PDH)), producing NADPH, which is quantified at 340/409 nm. Lactate is oxidized by lactate oxidase, generating a colored dye measured at 552/659 nm. Glutamine is deaminated and oxidized, forming a dye quantified at 552 nm, with a prior step eliminating glutamate interference. Glutamate undergoes oxidation, producing a dye measured at 552 nm. IgG is detected through an immunoturbidimetric assay, where precipitation with a specific antiserum is measured at 340 nm. All tests rely on photometric quantification.

### 2.3. In-Line Raman Spectra Acquisition

A Viserion Raman spectrometer (Indatech, Clapiers, France) was used for spectral acquisition. This features a 785 nm multimode fiber-coupled laser capable of delivering 500 mW of power at full capacity. The optical fibers used are low-OH Vis/NIR type, optimized for near-infrared applications, with a 220 µm quartz (SiO_2_) fiber core diameter and a polyimide coating. The detection system consists of a deep-cooled, back-illuminated charge-coupled device (CCD) with 2000 × 256 active pixels, each measuring 15 × 15 µm. The detector operates at −60 °C, providing a spectral resolution of 6 cm^−1^, which is interpolated to 1 cm^−1^ by Indatech’s proprietary software, MxVISERION (Version 1.06). The instrument is equipped with two probes. Each probe was connected to a stainless-steel immersion pipe, terminating in a tip equipped with a glass lens and a sapphire window. The working distance between the window and the focal plane was 0.5 mm. The pipes were disconnected from the probe heads for sterilization via autoclaving before use.

Shielding the bioreactor from daylight and artificial light was necessary. Without proper shielding, strong and sharp emission contributions appeared in the Raman spectra, exhibiting a variance higher than that of the chemical signals.

Throughout various batches, different acquisition times and numbers of scans were tested to prevent the CCD detector from saturating while maintaining a good signal-to-noise ratio. Among the five batches conducted, the optimal acquisition time was determined to be within the range of 9 to 15 min. For Batch 1, Raman acquisition parameters were set at 60 s per scan over 9 scans. For batches 2 and 3, this was followed with 10 scans of 90 s each. For Batches 4 and 5, the acquisition started with 16 scans of 45 s, followed by 9 scans of 75 s to increase the signal. Subsequently, the spectra intensity was normalized by dividing by the acquisition time. Detailed information on spectral preprocessing will be provided in the following section. The Viserion spectrometer’s spectral range was from 250 to 3200 cm^−1^, with a resolution of 1 cm^−1^. For each batch, Raman spectra were recorded continuously. Regardless of the acquisition time, a new Raman spectrum was recorded every 20 min (Batches 2, 3, 4, and 5) or 30 min (Batch 1), resulting in nearly 880 spectra on average per batch.

### 2.4. Chemometric Data Analysis

All chemometric analyses were performed using the PLS_Toolbox (Version 9.3) package (Eigenvector Research, Inc., Wenatchee, DC, USA) within the Matlab^®^ (R2023a) environment (The MathWorks, Inc., Natick, MA, USA).

To maximize the signal-to-noise ratio, three consecutive in-line Raman spectra were initially averaged and paired with their nearest offline reference measurements. Subsequently, different spectral preprocessing techniques were tested, including Savitzky–Golay smoothing, first and second derivatives, linear detrending, area normalization, and standard normal variate (SNV) to effectively correct the spectra by reducing baseline shifts, fluorescence, and inter-batch variability. The objective was to enhance the relevant signals for modeling [9,12]. In parallel, the spectral range was carefully selected to ensure relevance to analyte concentration changes. Finally, the spectra were mean-centered before proceeding with PLS modeling. The final selection of spectral range and preprocessing was based on the performance criteria of the PLS models.

Partial least squares (PLS) is a regression model used to correlate in-line Raman spectra with off-line reference values by identifying latent variables (LVs), which are underlying factors that summarize the information in the data. These LVs reduce the dimensionality of the spectra while maximizing covariance with the reference values. The PLS model is built using four batches (Batch 1, 3, 4, and 5, totaling 112 samples) with the largest estimated differences, and its performance is rigorously evaluated through 3-fold cross-validation. The root mean square error of cross-validation (RMSECV) helps to determine the ideal number of LVs for each regression model. Lastly, during an external validation phase, the remaining batch (Batch 2, totaling 31 samples) was used to assess the prediction accuracy of each PLS model. In addition, the coefficient of determination (R^2^) was calculated between the PLS model predictions and off-line reference measurements to evaluate model fit. Furthermore, the root mean squared prediction percentage error (RMSEP (%)) is used as a statistical measure for evaluating the accuracy of predictive models by comparing predicted values to observed values, expressed as a percentage of either the mean or the upper limit of the validation range.

In addition to the previously mentioned figures of merit, it is important to verify that the chemometric model is based on relevant variables reflecting the Raman bands of the analyte. The variable importance in projection (VIP) score is used as a metric in PLS regression models, especially for highly dimensional and multicollinear data. The VIP score quantifies the weight of each variable to the model’s performance. In practice, a VIP score greater than 1 indicates that the variable makes a significant contribution to the PLS regression model. Once identified, these selected variables can be interpreted and highlight their true contribution to the model.

Finally, the selected combinations (optimized final models) of preprocessing and variable selection are indicated in Section 3.

## 3. Results and Discussion

**Figure 2:**
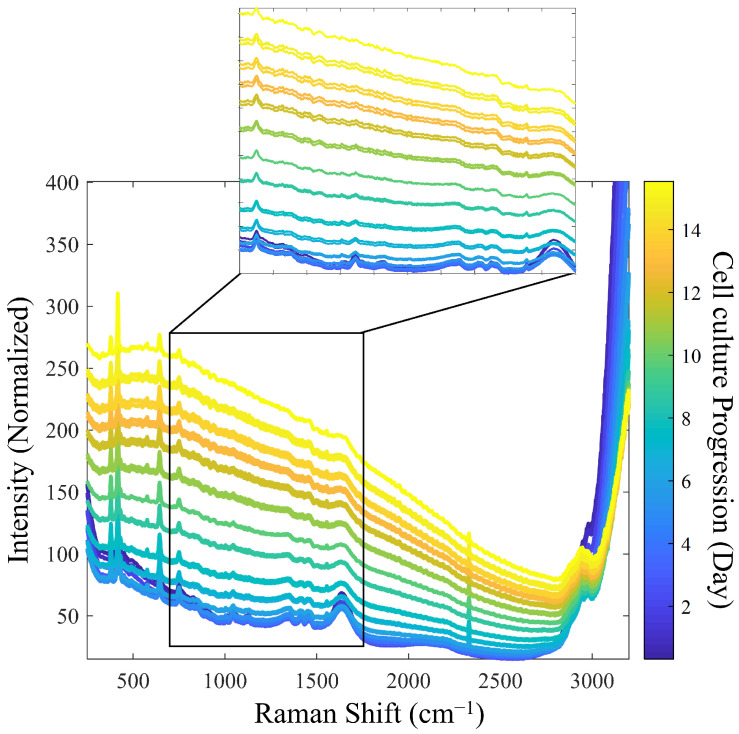
Evolution of Raman spectra during cell culture: detailed analysis of spectral changes from Batch 1 (initial stage: blue spectrum, final stage: yellow spectrum).

Figure 2 presents the reference Raman spectra utilized for model calibration. To improve the signal-to-noise ratio (SNR), spectral averaging was performed under the assumption that this would improve signal quality. However, due to the ongoing biological processes during the measurement period, excessive averaging would be counterproductive. As a result, an average of three spectra was performed.

During the progression of cell culture, significant changes in Raman spectra were observed. As expected, as the cell density increased, the turbidity-related background increased, accompanied by a decrease in the intensity of individual Raman bands, likely due to a decreased depth of light penetration in the increasingly turbid medium [5]. Specifically, the Raman bands of water from the culture medium, observed at 1640 cm^−1^ and 3100–3250 cm^−1^, diminished as the process progressed. In addition, other intensity decreases were observed for the bands related to the sapphire optical windows at 386, 424, and 645 cm^−1^ and for those related to dissolved oxygen (O_2_) and nitrogen (N_2_) at 1552 and 2331 cm^−1^, respectively [28].

Finally, the complexity of the cell culture medium, along with overlapping Raman bands from various analytes with similar chemical functional groups, complicates the isolation of distinct signals corresponding to nutrients and metabolites of interest [5,29]. For these reasons, chemometric analysis is essential to reveal hidden information in Raman spectra.

**Figure 3:**
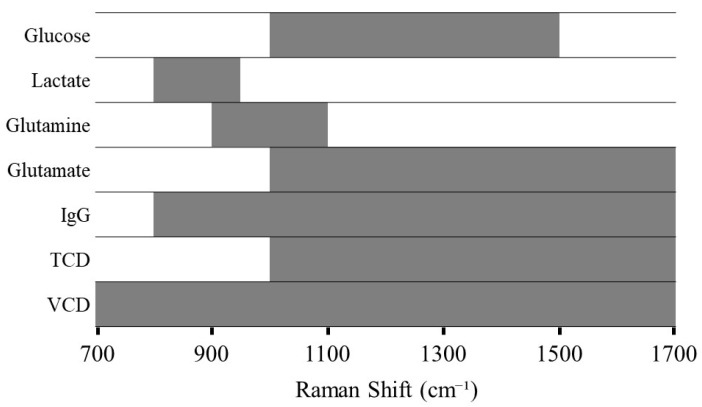
Graphical representation of selected spectral regions in PLS model development.

During the development of PLS models, various preprocessing techniques were tested (Figure S1), and the results consistently showed that specific algorithm combinations yielded better outcomes. Initially, derivative methods were applied to enhance spectral resolution and remove baseline, or smoothing algorithms were used to reduce noise. This was followed by area normalization or SNV transformation to minimize global intensity fluctuations between spectra. These combinations effectively enhanced the relevant signal and reduced inter-batch variability. For modeling each analyte, the selection of variables was focused on distinct spectral regions containing the relevant Raman bands, as painted in Figure 3. Thus, the spectral region between 700–1700 cm^−1^ was selected for further chemometric analysis, as it contains significant Raman bands corresponding to most of the molecules of interest, i.e., nutrients, metabolites, and IgG, as reported in the literature [5,29].

The quality of the spectra used in the models is of major importance, as the accuracy of any model is directly dependent on the quality of its input data-essentially, poor-quality input leads to unreliable results.

The integration time (Ti) and scan number (Nscan) were adjusted individually for the batches. The Ti allows to keep the signal strong enough but below the level when the spectra saturate, because of the turbidity-related background. Hence, in the more turbid samples, the Ti can be reduced to avoid saturation (see Section 2). In order to keep the spectral quality, the reduced Ti has to be compensated by increased Nscans. Since the Ti affects proportionally the spectra intensity, the spectra were normalized by dividing their intensity on the Ti, thus bringing all the spectral intensities to the equivalent one corresponding to 1 s acquisition. As a result of such pre-processing, there was no impact on the spectral quality. On the other hand, outliers have been eliminated.

The models were calibrated on four batches: three produced under normal operating conditions (Batches 1, 3, 4) and one batch with induced cell death to diminish cross-correlation (Batch 5). A total of 122 samples were placed in the calibration subset. The predictive performance of the models was evaluated using prediction errors, with a 3-fold cross-validation on the calibration subset and a blind test subset comprising one batch of 31 samples (Batch 2). Standard practices in PLS model development emphasize using a diverse range of information in calibration subsets to ensure the model’s robustness and specificity. Validation subsets, conversely, must remain independent from calibration subsets and should include the information the model will encounter during actual application. Additionally, determination coefficients (R^2^) were calculated to assess whether the Raman predictions and reference values were equal, ensuring good agreement in practice. The finalized PLS models are summarized in Table 1. As previously described, for each analyte a specific chemometric model has been developed with its own optimized pre-processing and variable selection protocol. Raman wavenumber selection (i.e., variables selection) was made based on two criteria: (i) their relevance as positions of Raman bands characteristic for a given molecule or parameter and (ii) their specific combination in case of similar band positions also present for other molecules or parameters.

### 3.1. PLS Models Associated with Nutrients, Metabolites, and IgG

**Figure 4:**
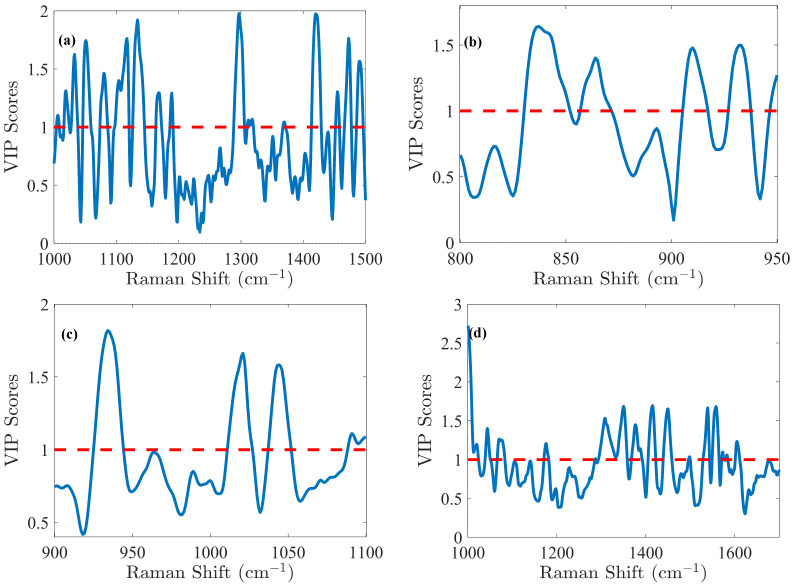

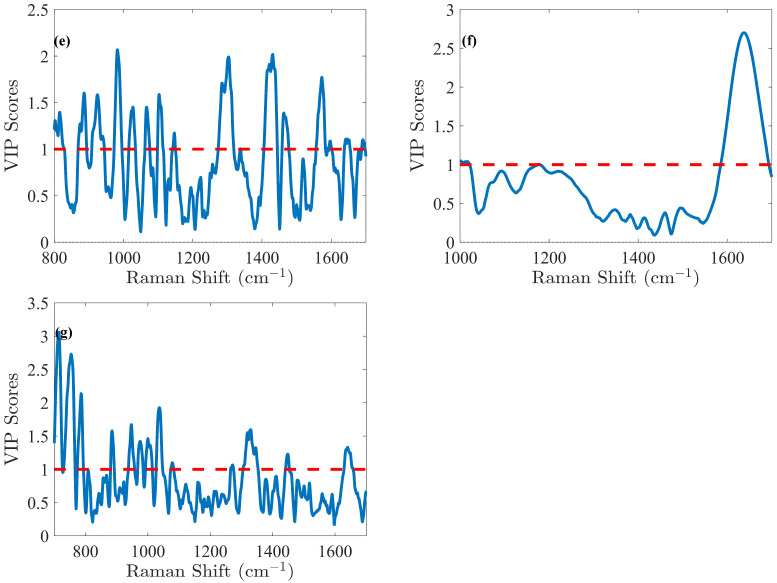
The VIP score obtained from the PLS model indicates the relative importance of the spectral region for (**a**) glucose (Glc), (**b**) lactate (Lac), (**c**) glutamine (Gln), (**d**) Glutamate (Glu), (**e**) IgG, (**f**) total cell density (TCD), and (**g**) viable cell density (VCD). These scores highlight the influence of each variable on the model’s predictive performance. VIP score > 1 means that the variable has a significant contribution.

The glucose (Glc) molecule is a six-membered ring (pyranose), which exists in equilibrium between its α- and β-forms. For the optimal PLS model of Glc, spectral data were preprocessed using the first derivative and SNV normalization, selecting the spectral range of 1000–1500 cm^−1^. The model was fine-tuned with 5 LVs via cross-validation, resulting in an RMSEC of 0.41 g/L and an RMSECV of 0.49 g/L. Calibration was performed across the range of 0.02 to 8.08 g/L. Validation resulted in an RMSEP of 0.51 g/L for the range of 2.14 to 7.17 g/L. The relative error RMSEP (%) was 10%, which is quite satisfactory for glucose predictions. These performance metrics demonstrate that the model is not overfitted and is robust, as indicated by the close agreement of error statistics. Glucose emerges as the analyte with the highest concentration in the cell culture, making it a straightforward candidate for model development. Notably, the VIP score (Figure 4a) method exhibits high sensitivity in regions associated with the glucose spectral fingerprint, identifying three critical spectral zones: 1000–1200 cm^−1^, a narrow band around 1250–1350 cm^−1^, and the range of 1400–1500 cm^−1^. Detailed analysis of the VIP score reveals prominent features within these regions, notably the C-O stretching at 1033 cm^−1^ (α) and 1060 cm^−1^ (β), along with COH bending at 1117 cm^−1^ [29]. Additionally, CH_2_ influences are detected with lower relative intensity, observed in wagging at 1318 cm^−1^ (α) and 1370 cm^−1^ (β), and bending at 1455 cm^−1^ [29].

The lactate (Lac) molecule is characterized by carboxyl, methyl, and hydroxyl groups. For this molecule, the PLS modeling was carried out in the 800–950 cm^−1^ spectral range. Preprocessing of the spectral data was carried out through the first derivative and SNV normalization. This optimized model yielded an RMSEC of 0.15 g/L, an RMSECV of 0.16 g/L, and an RMSEP of 0.19 g/L within a calibration range of 0.02–2.07 g/L and a validation range of 0.30–2.09 g/L, corresponding to an RMSEP (%) of 16%. In Figure 4b, the VIP score highlighted spectral contributions to PLS models from specific molecular vibrations, including C-COO^−^ (carboxylate) stretching between 830–852 cm^−1^, C-C stretching associated with COOH and COOCH functional groups from 857–872 cm^−1^, and C-H bending (β) functional groups between 905–917 cm^−1^ [29,30]. Additionally, CH_3_ rocking is observed in the 927–938 cm^−1^ region [29].

L-glutamine’s (Gln) amino acid structure consists of a central α carbon atom bonded to an amino group (–NH_3_^+^), a carboxylate group (-COO^−^), a hydrogen atom, and a side chain in the form of a γ-carboxyamide group (-CONH_2_). For the Gln modeling, the Raman spectra were processed using Savitzky–Golay smoothing. The PLS model, optimized with 6 LVs, demonstrated strong predictive capabilities. RMSEC was 0.39 mM, while RMSECV was 0.42 mM, indicating minimal overfitting. The model’s predictive performance, with an RMSEP of 0.53 mM, remained robust across the validation range of 0.19 to 4.82 mM, closely matching the calibration range of 0.20 to 4.83 mM (RMSEP (%) of 35%). The VIP score (Figure 4c) highlighted key spectral features: CH_3_ rocking at 934 cm^−1^ [31], C-CH_3_ stretching at 1021 cm^−1^ [31], C-O stretching (α) at 1044 cm^−1^ [31], and a region 1088–1100 cm^−1^ likely associated with NH_2_ rocking and C-O stretching (β) [31,32,33].

Similarly to Gln, L-glutamate (Glu) amino acid is also characterized by a central α-carbon bonded to an amino group (–NH_3_^+^), a carboxylate group (-COO^−^), but has a distinctive carboxyl side chain (-CH_2_-CH_2_-COO^−^). For the Glu modeling, the spectra were preprocessed using Savitzky–Golay smoothing, detrending, and standard normal variate (SNV) normalization to enhance batch comparability by minimizing batch effect variance. This approach yielded an RMSEC of 0.42 mM and an RMSEP of 0.65 mM within the calibration range of 0.73–11.18 mM, while the RMSEP reached 0.81 mM for a validation range of 0.76–10.12 mM, corresponding to an RMSEP (%) of 13%. According to the VIP score (Figure 4d), NH_2_ rocking was observed in the 1068–1083 cm^−1^ range [29], CH_2_ deformation between 1239–1358 cm^−1^ [34], and a complex mixture of COO^−^ symmetrical stretching, CH_2_ deformation, and symmetrical NH_3_⁺ deformation between 1405–1422 cm^−1^ [5,34,35].

For IgG titer, selecting six LVs established an efficient and robust predictive PLS model, while effectively preventing overfitting, which could distort predictions. The model’s predictive power is clearly demonstrated by plotting the measured reference antibody titers against the predicted concentrations, as illustrated in Figure S2e. The predictions closely align with the reference measurements, yielding a strong determination coefficient of 0.986 in cross-validation. Model calibration samples resulted in an RMSEC of 0.14 g/L and an RMSECV of 0.18 g/L within the 0.00–4.53 g/L calibration range. However, these errors, based solely on calibration samples, do not fully reflect the model’s true predictive abilities. To evaluate its full potential, the model was applied on an independent test subset, producing an RMSEP of 0.12 g/L across the validation range of 0.00–4.23 g/L, resulting in an RMSEP (%) of 8%, which is excellent considering the complexity of accurately predicting IgG concentration in a complex medium. For IgG, as for other proteins, Raman spectroscopy provides insights into both the protein primary structure (aromatic amino acids presence) and its secondary structure (amide bonds conformation), which generate distinct spectral patterns [29]. VIP score analysis (Figure 4e) further highlighted specific bands, including those associated with tryptophan (1554, 1595 cm^−1^) and tyrosine (1563 cm^−1^) [36] as well as the amide I region near 1643–1652 cm^−1^ [36,37], which is particularly significant.

### 3.2. PLS Models Associated with Cell Densities

Due to the chemical complexity of the culture medium, the concentrations of numerous analytes may evolve over time, either increasing or decreasing. Thus, a phenomenon known as cross-correlation can be observed, which can be expressed by a correlation coefficient (CC) between analytes. Its values can be positive or negative, indicating that the analytes either evolve in the same or opposite directions in terms of concentration. It ranges from –1 to 1, with values closer to either −1 or 1 indicating a stronger correlation between the analytes. In essence, cross-correlation helps assess how the levels of different analytes fluctuate in relation to each other. For example, changes in VCD are generally correlated with TCD, as long as cell death is negligible. When cross-correlation was computed between VCD and TCD for Batches 1 to 4 (cell cultures under normal operating conditions), high positive CC values were observed (>0.85). In contrast, Batch 5 (cell cultures with induced cell death) showed a lower CC of 0.34, representing a decrease of more than 60%. Thus, Batch 5 was useful to reduce the cross-correlations between VCD and TCD.

Concerning Total Cell Density (TCD), the optimal model was obtained by preprocessing the spectra using Savitzky–Golay smoothing and SNV normalization, focusing on the spectral range of 1000–1700 cm^−1^. This model delivered an RMSEC of 1.31 × 10^6^ cells/mL. Six LVs were selected through cross-validation, resulting in an RMSECV of 1.34 × 10^6^ cells/mL within the calibration range from 0.63 to 24.20 × 10^6^ cells/mL. Validation yielded an RMSEP of 1.01 × 10^6^ cells/mL, within a validation range from 0.70 to 24.30 × 10^6^ cells/mL, resulting in an RMSEP (%) of 7%. Analysis of the VIP score (Figure 4f) indicates that the spectral region 1600–1700 cm^−1^ is the primary contributor to the model. This region corresponds to the -OH band centered at 1640 cm^−1^, commonly associated with water. As explained above, it is unsurprising that this region plays a dominant role, as it is notably affected by cell culture medium turbidity (e.g., during cell growth). In the case of TCD, the efficiency of PLS models in this context is attributed to the influence of turbidity, which alters the shape of the Raman spectra. Thus, with progressing cell culture, cell densities increase, including both live cells and the remnants of dead cells. This rise causes higher nominal intensity values due to scattering, the effect of which could be indirectly linked to cell densities and reflecting ongoing biological changes. Despite this, the -OH band remains valuable for tracking process evolution. Indeed, cells alone do not exhibit distinct Raman signals [5]. In Figure S2f, the reference TCD values are plotted against the PLS-predicted TCD values. The R^2^ in calibration demonstrated a strong correlation, confirming linearity (R^2^ > 0.95). However, at higher TCD values, a plateau emerged, clearly indicating that predicted values for higher TCD values remained nearly constant. This unequivocally implies a non-linear Raman signal of the water band in the later stages of cell culture. Indeed, despite a decrease in Raman signal intensity in the water band, this reduction occurs in a non-linear way as the culture progresses and could be due to the turbidity of the culture medium. Finally, while the model holds potential for industrial application, its specificity is likely to be process-dependent, particularly in relation to turbidity and its overall impact on the signal.

To optimize the PLS model for Viable Cell Density (VCD), different preprocessing methods and spectral ranges were evaluated in comparison with TCD. The optimal preprocessing approach identified was to use a second derivative followed by area normalization. The selected spectral range for analysis was from 700 to 1700 cm^−1^. Cross-validation determined that five LVs were optimal for the PLS model. An RMSEC value of 1.32 × $10^{6}$ cells/mL and an RMSECV value of 1.53 × $10^{6}$ cells/mL were obtained within the calibration range of 0.42 to 22.80 × $10^{6}$ cells/mL. RMSEP was determined to be 1.71 × $10^{6}$ cells/mL, within a validation range spanning from 0.69 to 22.80 × $10^{6}$ cells/mL, equivalent to an RMSEP (%) of 12%. The VIP coefficients were calculated for each variable to assess their significance within the model. Unlike the TCD model, the important variables for the VCD model are dispersed across the entire Raman spectrum, showing that other spectral regions contribute significantly to the model. Figure 4g illustrates the VIP score derived from the model. Peaks at 782–783 cm^−1^ are attributed to nucleic acids, the peak at 811 cm^−1^ is associated with RNA, more specifically, the O–P–O stretching mode of the sugar-phosphate backbone, but possibly also the ring breathing vibration of the tyrosine amino acid, and the peak at 1100 cm^−1^ corresponds to DNA [38]. The peak at 702 cm^−1^ is indicative of cholesterol, while peaks at 717 cm^−1^ and 1450 cm^−1^ are related to lipids. Additionally, the peak at 725 cm^−1^ is assigned to adenine. As previously stated, cells alone do not exhibit distinct Raman signals [5]. Thus, in the case of VCD, a possible hypothesis could be that during cell death, chemical and biological materials are released into the cell culture medium, directly contributing to nucleic acid-, protein-, and lipid-related Raman bands, which are thus indirectly associated with VCD [39].

### 3.3. Overview of PLS Regression Models Performance

Overall, during the development of PLS regression models, exploring different preprocessing methods as well as variable selection was useful when modeling chemical information. Those approaches facilitated the extraction of relevant spectral information and ensured consistency across different batches. After the development of PLS modeling, the resulting VIP scores indicate the most contributive spectral regions to the models, underscoring the analyte-specific nature of each PLS model. As the spectral areas are different for different parameters, this reinforces the specificity of the models. Additionally, the relatively high number of LVs, ranging between four and eight, suggests that the complexity of the analytes requires a more intricate modeling approach for accurate PLS predictions. Additionally, for each model, the analysis of standardized residuals vs. leverage plots (Figure S3) demonstrates that there are no outliers in our models. A few points with high leverage but low residuals are influential yet well-fitted, while points with low leverage but high residuals, despite indicating poor prediction accuracy, do not significantly impact the regression model.

In biotechnological processes involving CHO cells, monitoring at least glucose, lactate, and IgG is imperative: glucose serves as the primary carbon source, lactate accumulation is a toxic byproduct that negatively impacts cell viability and metabolic conditions, and IgG is the target molecule, making its yield a primary measure of process productivity and final application relevance [40,41]. Therefore, it is important to contextualize PLS regression models within the framework of the bioprocess. The RMSECV error values for the various PLS models must be evaluated not only against the requirements for bioreactor monitoring but also in comparison to the precision and accuracy of the reference methods on which the modeling is based. In our experience, standard errors of off-line reference concentrations measured using a Roche Cedex Bio HT (for substrate and metabolite quantification) and Beckman Coulter ViCell (for cell density quantification) may reach 10–15%. Predicted values derived from Raman measurements, therefore, inherently reflect these uncertainties and are unlikely to surpass this precision.

**Figure 5:**
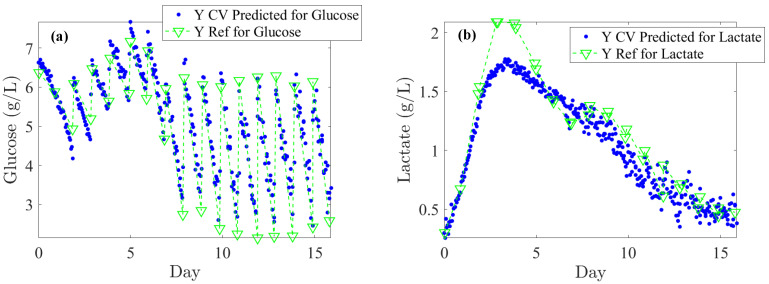

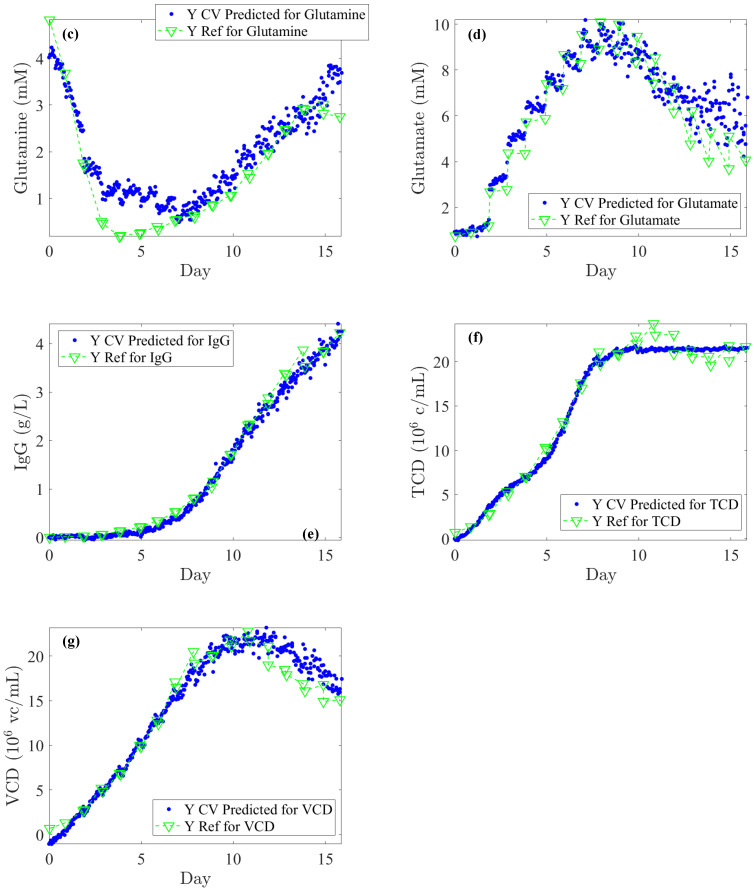
Once PLS models are optimized on Batches 1, 3, 4, and 5, they are used on the test subset (Batch 2) for back-prediction: comparison of off-line reference values (blue dots) and Raman spectra-based predictions (green lines and downward-pointing triangle) for: (**a**) Glucose, (**b**) Lactate, (**c**) Glutamine, (**d**) Glutamate, (**e**) IgG, (**f**) TCD (Total Cell Density), and (**g**) VCD (Viable Cell Density).

The literature generally indicates that glucose levels must be maintained above 1 g/L. In our off-line reference values, rapid depletion of glucose is observed over 24 h periods, dropping from 6 to 2 g/L (see Figure 5a). Considering process requirements, glucose levels could be regulated at an average of 5 ± 0.5 g/L. An RMSECV (%) of 12.8% supports this target as both feasible and satisfactory in terms of performance. For lactate concentration, a toxicity threshold of 2 ± 0.2 g/L should trigger alerts at this level. Consequently, an RMSECV (%) of 7.8% based on the upper limit is suitable for effective CHO culture management. Furthermore, monitoring IgG titer across all concentrations is essential to closely track cell productivity and process efficiency. Based on the instrument’s standard error, an RMSECV (%) of 9.3% is acceptable. Accordingly, as shown in Table 1, the RMSEC, RMSECV, and RMSEP errors meet the minimum required thresholds. Specifically, for glucose, the error remains consistently below 0.5 g/L; for lactate, it remains below 0.2 g/L across RMSEC, RMSECV, and RMSEP; and for IgG, the error stays below 0.1 g/L for both RMSEC and RMSEP.

When evaluating the robustness of PLS regression models on blind test subsets, the average RMSEP (%) errors for the substrates were found to be 10.2% for glucose and 34.7% for glutamine. For IgG titer, the RMSEP (%) was 8.1%, while for cell densities, values were found to be 6.6% for TCD and 2.2% for VCD. RMSEP (%) errors based on the upper limit were 9.0% for lactate and 8.0% for glutamate. Given an acceptable error threshold of 15%, these results demonstrate that the model provides satisfactory accuracy for glucose, lactate, glutamate, IgG titer, TCD, and VCD. In particular, developing calibration models for glutamine appears to be more challenging due to its low concentrations, the weak intensity of their peaks, and their overlap with peaks from other components. This aligns with previous findings [4,29,35].

The scope of this study focused on monitoring; however, it is important to note that these errors hold broader relevance within the process analytical technology framework. In fact, the next step could involve controlling a pump to selectively add nutrients as needed.

Lastly, Figure 5 illustrates the Raman back-predictions based on the test subset (Batch 2) compared to off-line reference values. The comparison of these two curves highlights their strong agreement and demonstrates the potential to accurately describe the evolution of nutrients, metabolites, IgG titer, and cell densities over the course of a cell culture.

## 4. Conclusions and Perspectives

In this study, Raman spectroscopy was employed to monitor nutrients, metabolites, IgG titer, and cell densities during five distinct 10 L-scale cell cultures. PLS regression models were calibrated using four batches, three of which followed normal operating procedures, while one batch was intentionally subjected to induced cell death to reduce cross-correlation between analytes. This strategy proved to favor the robustness of the PLS regression models. A strong squared correlation was observed between the predicted and reference values across the blind test subsets, with R^2^ values exceeding 0.9, confirming the predictive accuracy of the models. Model validation through comparison of calibration and validation errors (RMSEC vs. RMSEP) demonstrated the models’ robustness, with similar error values observed. Notably, glucose and IgG titer showed RMSEC and RMSEP values of 0.41 g/L vs. 0.51 g/L and 0.14 g/L vs. 0.12 g/L, respectively. Lactate also showed an RMSEP (%) of 9.0%, within an acceptable range for biological variability. However, the analysis also revealed higher RMSEP (%) values for some analytes, suggesting model limitations in specific cases. For glutamine within the validation range 0.19–4.82 mM, the RMSEP (%) reached 34.7%, which is considerably high and points to a potential lack of robustness, undoubtedly due to its low concentration. Analysis of the VIP score highlighted the contributions of Raman shifts from glucose, lactate, glutamine, glutamate, and IgG to the models, consistent with specificity. However, specificity was not proven for TCD and VCD, likely due to the absence of a Raman signal specific to the cells. Nonetheless, for TCD, the region around 1640 cm^−1^, linked to water bands and turbidity, was a significant contributor, while for VCD, the model’s predictions were indirectly influenced by bands related to nucleic acids, proteins, and lipids.

These results are promising, as this real-time, non-invasive Raman spectroscopy monitoring represents a significant breakthrough in bioprocess control. The results demonstrate that cell culture status can be monitored in under 12 min without sampling. The VIP score further improves model interpretability by highlighting the contributions of individual variables to the performance of PLS models. Finally, this approach opens the door to automation in bioprocess control. By integrating online Raman spectrometry into a feedback control system, it could be possible to actively regulate key nutrient concentrations, such as glucose, through continuous feeding and to maintain predefined setpoints to improve critical quality attributes (CQAs).

## Figures and Tables

**Table 1:** Summary of PLS model performance optimized through variable selection and spectral preprocessing techniques [D1 (width, order) and D2 (width, order) represent the first and second derivatives with Savitzky-Golay smoothing; SG (width, order) is Savitzky–Golay smoothing without derivatives; width defines the number of points for the polynomial, and order refers to its degree; SNV stands for standard normal variate].

	Preprocessing				Calibration Subset		Cross-Validation Set		Test Subset			
		Var. Sel.	LVs	Cal. Range	R^2^	RMSEC	R^2^	RMSECV	Val. Range	R^2^	RMSEP	RMSEP (%)
Glucose (g/L)	D1 (23, 2) + SNV	1000:1500	5	0.02–8.08	0.97	0.41	0.96	0.49	2.14–7.15	0.93	0.51	10.2 (average)
Lactate (g/L)	D1 (35, 2) + SNV	800:950	4	0.02–2.07	0.94	0.15	0.93	0.16	0.30–2.09	0.94	0.19	9.0 (max)
Glutamine (mM)	SG (23, 2)	900:1100	6	0.20–4.83	0.85	0.39	0.83	0.42	0.19–4.82	0.91	0.53	34.7 (average)
Glutamate (mM)	SG (35, 2) + Detrend + SNV	1000:1700	8	0.73–11.18	0.97	0.42	0.93	0.65	0.76–10.12	0.93	0.81	8.0 (max)
IgG (g/L)	D1 (55, 2) + SNV	800:1700	6	0.00–4.53	0.99	0.14	0.99	0.18	0.00–4.23	1.00	0.12	8.1 (average)
TCD (10^6^ cells/mL)	SG (45, 2) + SNV	1000:1700	6	0.63–24.20	0.96	1.31	0.95	1.34	0.70–24.30	0.98	1.01	6.60 (average)
VCD (10^6^ cells/mL)	D2 (81, 2) + Normalize (1-Norm. Area = 1)	700:1700	5	0.42–22.80	0.96	1.32	0.94	1.53	0.69–22.80	0.95	1.71	12.2 (average)

## Data Availability

The datasets presented in this article are not readily available because they consist of proprietary industrial data that cannot be shared.

## Supplementary Materials

The following supporting information can be downloaded at: https://www.mdpi.com/article/10.3390/pharmaceutics17040473/s1. Figure S1: Representation of the effects of spectral preprocessing on Raman spectra calibration subsets for Batches 1, 3, 4, and 5; Figure S2: Raman predictions vs. measured off-line reference: evaluating PLS model performance using calibration data set of 122 samples (Batches 1, 3, 4, and 5); Figure S3: Standardized residuals vs. leverage plots to identify influential data points.

## Abbreviations

The following abbreviations are used in this manuscript:

Cal. Range	Calibration Range
CCD	Charge-Coupled Device
CHO	Chinese Hamster Ovary
CNNs	Convolutional Neural Networks
CPPs	Critical Process Parameters
CQAs	Critical Quality Attributes
D1	First Derivative
FDA	Food and Drug Administration
Glc	Glucose
Gln	Glutamine
Glu	Glutamate
IgG	Immunoglobulin G
Lac	Lactate
LVs	Latent Variables
mAb	Monoclonal Antibody
NADPH	Nicotinamide Adenine Dinucleotide Phosphate (Reduced)
PAT	Process Analytical Technology
PLS	Partial Least Squares
R^2^	Coefficient of Determination
RF	Random Forest
RMSEC	Root Mean Square Error of Calibration
RMSECV	Root Mean Square Error of Cross-Validation
RMSEP	Root Mean Square Error of Prediction
RMSEP (%)	RMSEP as a Percentage
SG	Savitzky–Golay Smoothing
SNR	Signal-to-Noise Ratio
SNV	Standard Normal Variate
SVM	Support Vector Machine
Val. Range	Validation Range
Var. Sel.	Variable Selection
VCD	Viable Cell Density
