# Supplementary material for: Monitoring of Nutrients, Metabolites, IgG Titer, and Cell Densities in 10 L Bioreactors Using Raman Spectroscopy and PLS Regression Models
Source: Pharmaceutics. 2025 Apr 4;17(4):473. doi: 10.3390/pharmaceutics17040473 (PMC12030344; doi:10.3390/pharmaceutics17040473)
Supplement: Supplementary file 1 [file pharmaceutics-17-00473-s001.zip › pharmaceutics-3527169-supplementary.pdf]

Supplementary Materials

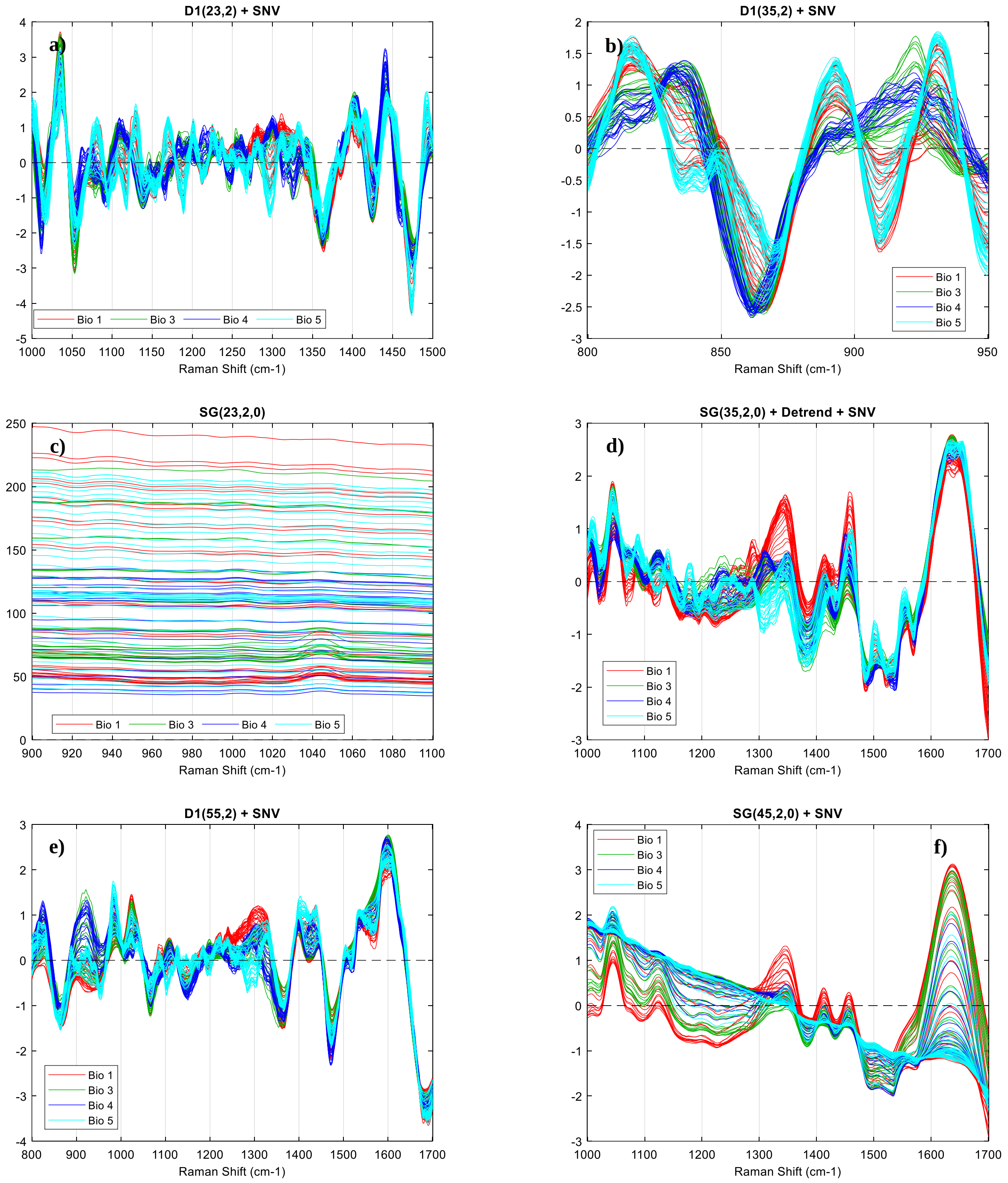

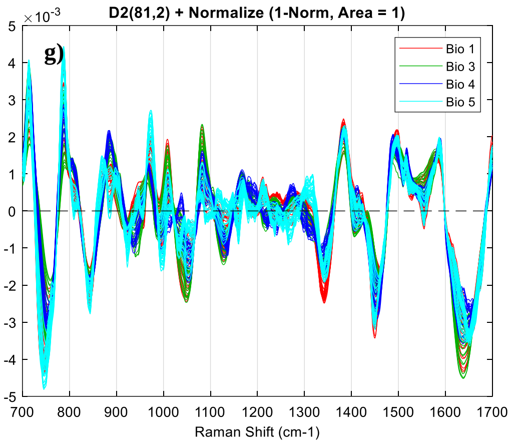

Figure S1. Representation of the effects of spectral preprocessing on Raman spectra calibration subsets for Batches 1, 3, 4, and 5. a) Glucose (1st derivative (width: 23, order: 2) + SNV), b) Lactate (1st derivative (width: 35, order: 2) + SNV), c) Glutamine (Savitzky-Golay, width: 23, order: 2), d) Glutamate (Savitzky-Golay, width: 35, order: 2 + Detrend + SNV), e) IgG (1st derivative (width: 55, order: 2) + SNV), f) Total Cell Density (Savitzky-Golay, width: 45, order: 2 + SNV) and g) Viable Cell Density (2nd derivative (width: 81, order: 2) + Area Normalization)

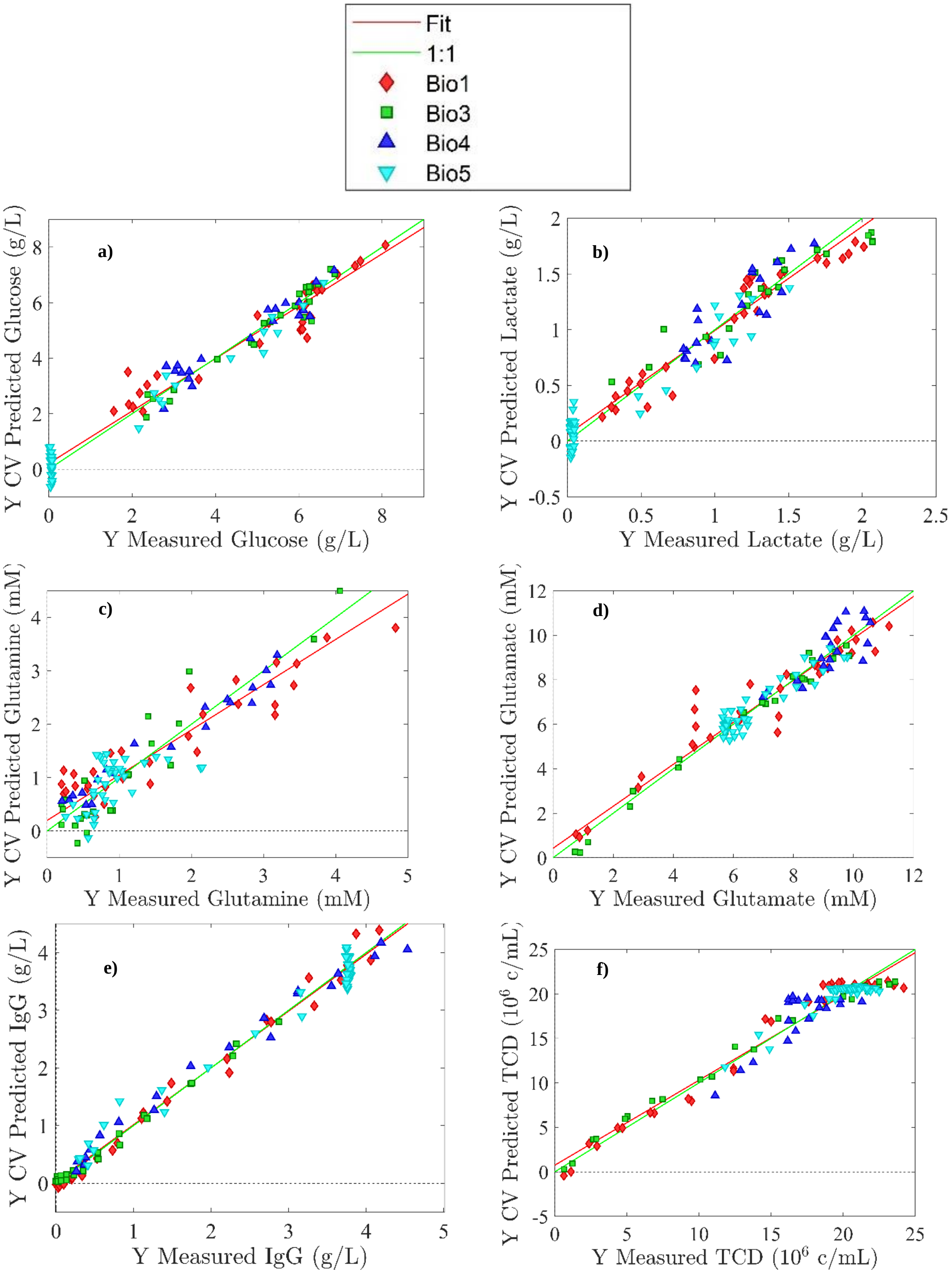
Fit
1:1
Bio1
Bio3
Bio4
Bio5
a)
Y CV Predicted Glucose (g/L)
Y Measured Glucose (g/L)
b)
Y CV Predicted Lactate (g/L)
Y Measured Lactate (g/L)
c)
Y CV Predicted Glutamine (mM)
Y Measured Glutamine (mM)
d)
Y CV Predicted Glutamate (mM)
Y Measured Glutamate (mM)
e)
Y CV Predicted IgG (g/L)
Y Measured IgG (g/L)
f)
Y CV Predicted TCD (10^6 c/mL)
Y Measured TCD (10^6 c/mL)

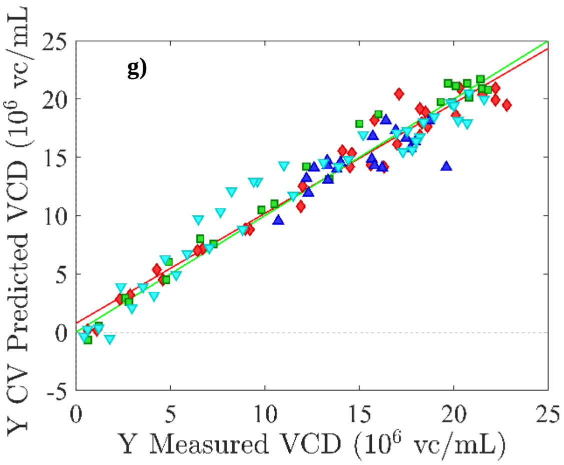

Figure S2. Raman predictions vs. measured *off-line* reference: evaluating PLS model performance using calibration data set of 122 samples (Batches 1, 3, 4, and 5). Green lines represent bisectors, red lines indicate actual PLS model performance for: a) Glucose, b) Lactate, c) Glutamine, d) Glutamate, e) IgG, f) TCD (Total Cell Density), and g) VCD (Viable Cell Density). The legend associated with the figures is as follows:

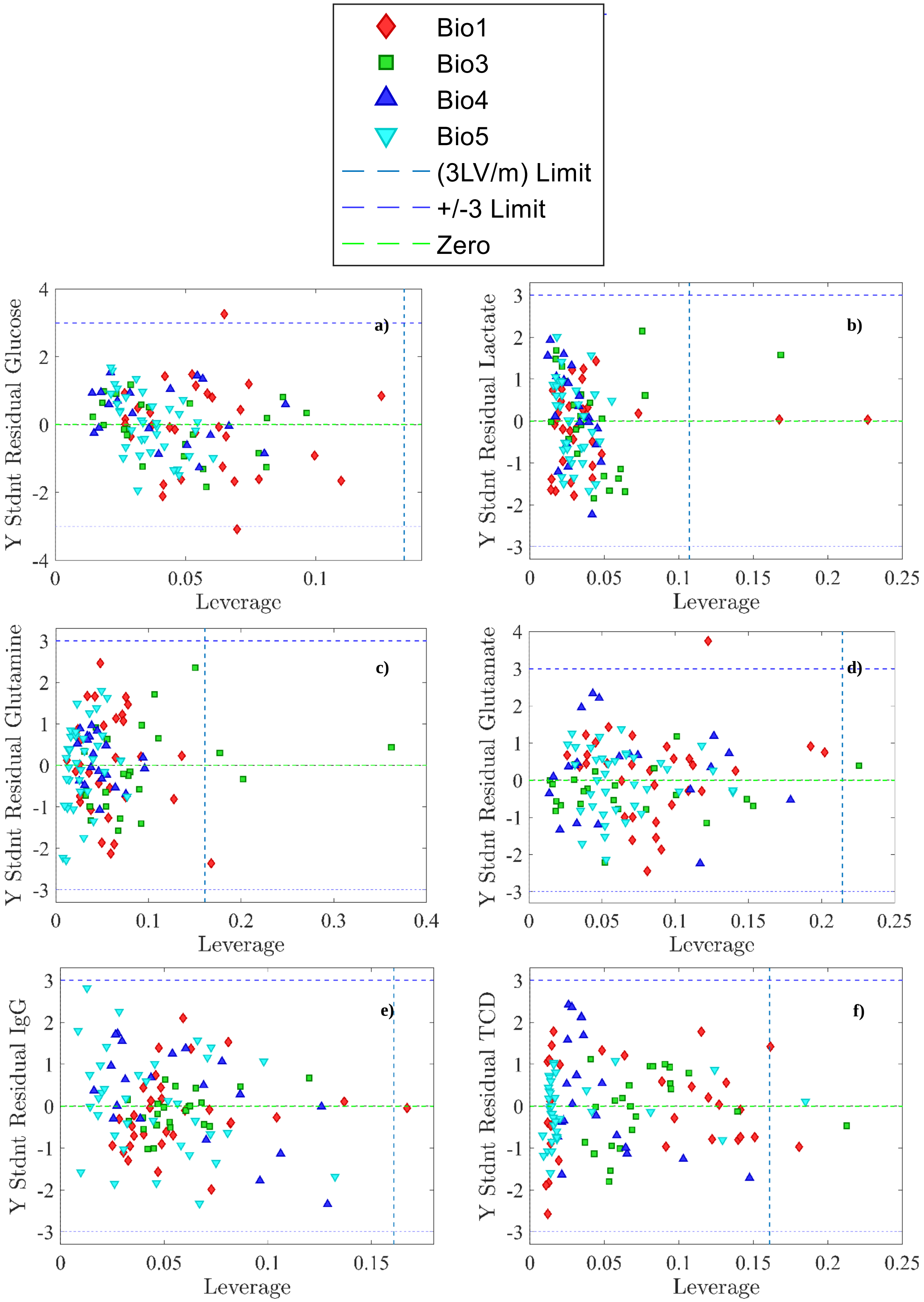
Bio1
Bio3
Bio4
Bio5
(3LV/m) Limit
+/-3 Limit
Zero
a)
Y Stdnt Residual Glucose
Leverage
b)
Y Stdnt Residual Lactate
Leverage
c)
Y Stdnt Residual Glutamine
Leverage
d)
Y Stdnt Residual Glutamate
Leverage
e)
Y Stdnt Residual IgG
Leverage
f)
Y Stdnt Residual TCD
Leverage

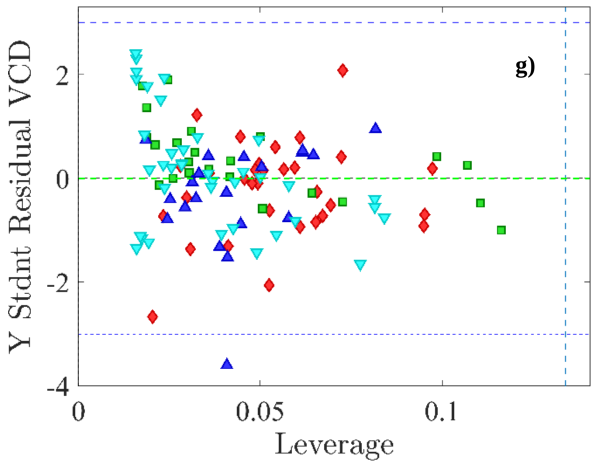

Figure S3. The figure presents standardized residuals vs. leverage plots for: a) Glucose, b) Lactate, c) Glutamine, d) Glutamate, e) IgG, f) TCD (Total Cell Density), and g) VCD (Viable Cell Density). The vertical axis shows standardized residuals, which measure the discrepancy between observed and predicted values after standardization: high values indicate atypical residuals or outliers. The horizontal axis represents leverage, quantifying the potential influence of a point on the regression: high leverage values indicate points that are significantly distant from the predictor mean. Conventionally, points near the origin are considered unproblematic, while points with high leverage but low residuals are influential yet well-fitted. In contrast, points with both high leverage and high residuals are problematic, as they heavily influence the model while fitting poorly. Finally, points with low leverage but high residuals are outliers that, despite showing poor prediction accuracy, do not substantially impact the regression model. For glucose, two points from Bio1 reached the standardized residual limits but showed low leverage. For lactate, three points (two from Bio1 and one from Bio3) exhibited high leverage without high standardized residuals. Similarly, for glutamine, four points (one from Bio1 and three from Bio3) had high leverage but low standardized residuals. In the case of glutamate, one point from Bio1 displayed a high standardized residual but low leverage, while one point from Bio3 had high leverage but a low standardized residual. For IgG, one point showed high leverage. Regarding TCD, two points from Bio1, one from Bio5, and one from Bio3 had high leverage with low standardized residuals, while for VCD, one point from Bio4 showed a high residual. All these data points are retained for subsequent data analysis to challenge the models during the calibration phase. The legend associated with the figures is as follows:
